# Lysine methylation of transcription factors in cancer

**DOI:** 10.1038/s41419-019-1524-2

**Published:** 2019-03-29

**Authors:** Dong Han, Mengxi Huang, Ting Wang, Zhiping Li, Yanyan Chen, Chao Liu, Zengjie Lei, Xiaoyuan Chu

**Affiliations:** 1 Department of Medical Oncology, Jinling Hospital, Nanjing Clinical School of Southern Medical University, Nanjing, Jiangsu Province China; 2 Department of Medical Oncology, Jinling Hospital, Medical School of Nanjing University, Nanjing, Jiangsu Province China; 3 Department of Medical Oncology, Jinling Hospital, Nanjing Clinical School of Nanjing Medical University, Nanjing, Jiangsu Province China

## Abstract

Protein lysine methylation is a critical and dynamic post-translational modification that can regulate protein stability and function. This post-translational modification is regulated by lysine methyltransferases and lysine demethylases. Recent studies using mass-spectrometric techniques have revealed that in addition to histones, a great number of transcription factors are also methylated, often at multiple sites and to different degrees (mono-, di-, trimethyl lysine). The biomedical significance of transcription factor methylation in human diseases, including cancer, has been explored recently. Some studies have demonstrated that interfering with transcription factor lysine methylation both in vitro and in vivo can inhibit cancer cell proliferation, thereby reversing tumor progression. The inhibitors targeting lysine methyltransferases and lysine demethylases have been under development for the past two decades, and may be used as potential anticancer agents in the clinic. In this review, we focus on the current findings of transcription factor lysine methylation, and the effects on both transcriptional activity and target gene expression. We outlined the biological significance of transcription factor lysine methylation on tumor progression and highlighted its clinical value in cancer therapy.

## Facts

1. Abnormal transcriptional activity is an important part of tumorigenesis.
2. The activity of transcription factors is regulated by post-translational modifications, especially lysine methylation.
3. Several protein lysine methyltransferase inhibitors have been proven as promising new targets for anticancer therapy.

## Open questions

1. Lysine methylation of transcription factors has been discovered in recent years. What role does this post-translational modification play in cancer?
2. What is the specific mechanism of lysine methylation in regulating transcription factor activity?
3. Epigenetics provides promising new targets for anticancer therapy. Does targeting lysine methylation of transcription factors provide important clinical value?

## Introduction

Transcription factors are a group of proteins that can bind to specific sequences upstream of the 5′ terminus of target genes, typically considered the promoter region^1,2^. In this way these transcription factors can inhibit or enhance gene expression and ensure specific temporal target gene expression^3^. Under normal circumstances, promoter-specific transcription factors contribute in basic biological activities including differentiation^4^, development^5^, and metabolism^6^. Importantly, dysregulation of these transcriptional programs can lead to malignant growth and cancer formation^7,8^. Transcription factors can be subject to a variety of enzyme-catalyzed post-translational modifications (PTMs) in response to environmental changes, especially in disease occurrence and tumorigenesis^9,10^.

These transcription factor PTMs are added and removed by the same enzyme families that are involved in histone modifications like acetylation, phosphorylation, and methylation^11–13^. Specific modifications have selective effects on transcription factor functions, resulting in specific gene expression alterations. It has been demonstrated in the literature that transcription factor phosphorylation and acetylation can promote carcinogenesis by regulating transcriptional activity^14,15^. We have greatly improved our understanding of transcription factor methylation with the development of mass-spectrometric techniques in the last few decades^16^.

Protein methylation occurs at specific sites on substrates, with lysine methylation being one of the important forms^17–19^. The lysine (K) ε-amino group of protein substrates can accept up to three methyl groups, resulting in either mono-, di-, or trimethyl lysine, in a process termed lysine methylation^20–22^. Recent studies have revealed that a number of transcription factors have been found to be modified by lysine methyltransferases (KMTs)^23–25^, resulting in specific gene expression alterations^26,27^. The abnormal expression of methyltransferases in many tumor types, which has been proven to be associated with tumorigenesis and cancer development, has become the focus of anticancer research^28–30^. In addition to histone methylation^31^, transcription factor methylation modification is also an important aspect for the development of cancer^27,32^.

To date, multiple studies have demonstrated that lysine methylation of transcription factors can directly regulate target gene expression by altering transcription factor stability and function. In this review, we summarize recent studies on lysine methylation of transcription factors, aiming to underline the biological significance and highlight the potential clinical value of lysine methylation of transcription factors in cancer.

## The process of protein lysine methylation

**Fig. 1:**
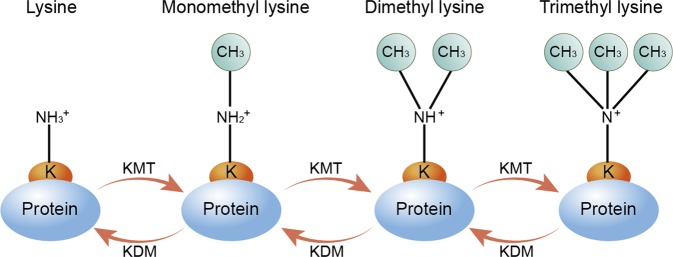
The process of lysine methylation and demethylation. Lysine (K) methylation is a dynamic and reversible post-translational modification (PTM) of proteins. Generally, the lysine ε-amino groups can accept up to three methyl groups, resulting in mono-, di-, or trimethyllysine. Lysine methyltransferases (KMTs) catalyze the addition of methyl groups to substrates, while lysine demethylases (KDMs) remove methyl groups. K, lysine; PTM, post-translational modification; KMTs, lysine methyltransferases; KDMs, Lysine methyltransferases

The process of protein lysine methylation consists of enzymes adding or removing methyl groups on particular substrates^33,34^ (Fig. 1). The lysine ε-amino group of proteins can accept up to three methyl groups, resulting in either mono-, di-, or trimethyl lysine, (me1, me2, or me3) with the various methylation states of lysines exerting distinct functions^35^. To date, more than 50 KMTs and 20 lysine demethylases (KDMs) have been reported^36^.

## Lysine methyltransferases

The lysine methyltransferases that methylate histones can also methylate non-histone proteins^36^, which have been categorized into eight classes according to their sequences and structures. The two largest classes are the SET proteins, containing a defined SET-domain, and the seven-β-strand (7BS) proteins, which have a typical core fold of seven strands^25^. The SET-domain proteins mostly target the lysines in the flexible tails of histones. In general, the lysine ε-amino group can accept up to three methyl groups, resulting in either mono-, di-, or trimethyl lysine, with the different methylation states of lysine exerting distinct functions^35^. In contrast, the majority of 7BS KMTs target a single protein or a group of highly related proteins. Complete understanding of the functional consequences of methylation of 7BS KMT targets still remains elusive, and in most cases the relationship between biological functionality and the biochemistry is challenging to understand^37^.

## Lysine demethylases

Lysine methylation had historically been considered irreversible until the first histone demethylase, Lys-specific demethylase 1 (LSD1, also known as KDM1A, BHC110, and AOF2), was discovered in 2004^38,39^. LSD2 (also known as KDM1B) is the only homolog of LSD1 in the human genome. LSD1 and LSD2 both belong to the first KDM family of flavin-dependent monoamine oxidases, and only demethylate monomethyl and dimethyl lysine residues^40^. The second family of KDMs consist of Jumonji C (JMJC) domain-containing proteins^41^, which use an oxygenase mechanism to demethylate monomethylated, dimethylated, and trimethylated lysine residues^18^.

## Substrates

Since the discovery of protein methylation more than 50 years ago^42^, most studies have focused on histone methylation in epigenetic domains^43^. However, with the development of mass-spectrometric techniques, there has been extensive broadening of our understanding of known PTMs and the corresponding protein targets^16,44,45^. Recent developments in protein mass spectrometry have allowed for high-throughput identification of lysine-methylated proteins, and nearly 2000 methyl modifications on lysine residues, distributed roughly between 1200 different proteins, have been reported in the human proteome. However, the biological function of the majority of these methylations still awaits identification^37^.

Many of the dynamic changes in gene expression that occur in response to extracellular signals are mediated by PTMs that regulate the activity of promoter-specific transcription factors^46,47^. Lysine methylation is emerging as an important regulatory mechanism of transcription factor function, where alteration of this modification activates or represses gene expression. The biomedical significance of non-histone lysine methylation, including of transcription factors, in several human diseases has been explored in recent years^48,49^.

## Regulatory mechanisms of lysine methylation

**Fig. 2:**
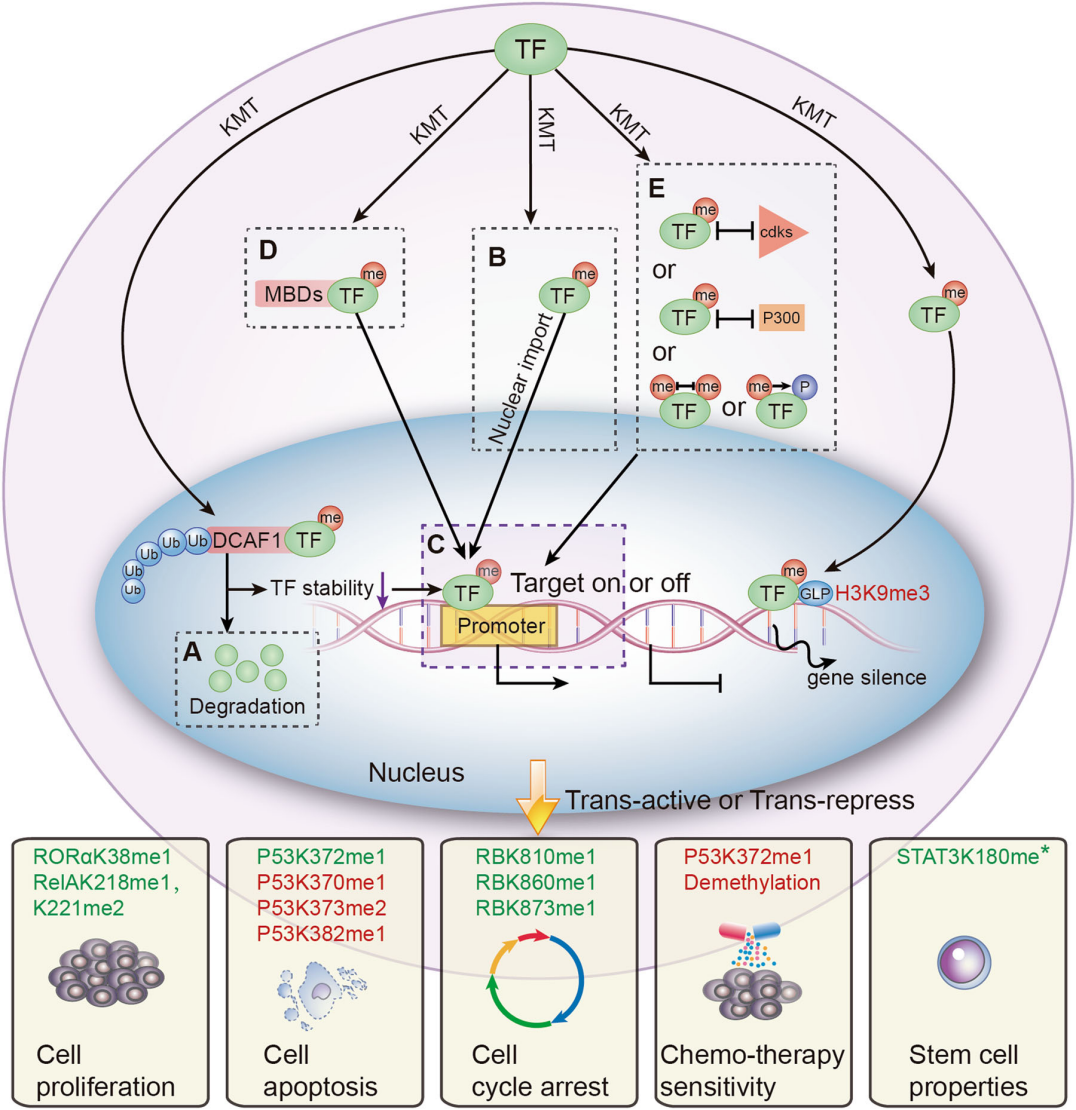
The regulation mechanisms of lysine methylation of transcription factors and the downstream effects on cell biology. Lysine methylatioin can regulate transcription factor (TF) function by altering protein stability, subcellular localization, DNA-binding affinity, protein–protein interactions, and crosstalk with other PTMs. Notably, lysine methylation modifications may alter cellular biological processes positively (in green) or negatively (in red). **a** Protein stability can be regulated by lysine methylation indirectly. **b** Like phosphorylation, lysine methylation can also alter nuclear localization of transcription factors, thus regulating transcriptional activity. **c** Lysine methylation positively or negatively regulates promoter binding affinity of transcription factors, thereby altering transcription of target genes. **d** Lysine methylation can affect protein–protein interactions. Methylated lysine can be recognized by proteins that contain special motifs such as chromo, tudor, or malignant brain tumor domains, resulting in differential biological effects. **e** Lysine methylation regulates other PTMs in the same or adjacent sites. TF, transcription factor; me, methyl group; ac, acetyl group; P, phosphate group; Ub, ubiquitin; DCAF1, DDB1-CUL4-associated factor 1; MBD, methylation-binding domain; p300, lysine acetyltransferase p300; GLP, histone methyltransferase GLP; *the methylation status is unknown

Many review articles have focused on the various effects of transcription factor phosphorylation^15,50^, SUMOylation^51^, ubiquitination^52^, acetylation^14^, and glycosylation^10^. Like other PTMs, protein lysine methylation can directly regulate distinct aspects of transcription factor function, including protein stability, cellular localization, DNA-binding affinity, protein–protein interactions, and crosstalk with other PTMs. Although some lysine methylation phenomena are observed in some cases, the specific regulatory mechanisms still remain to be clarified^53–56^. Herein we discuss the five major regulatory mechanisms of lysine methylation based on the current literature (Fig. 2 and Table 1).

**Table 1:** The main regulatory mechanisms of transcription factor lysine methylation

Mechanism	Substrate^a^	Enzyme^b^	Tumor type	Transcription activity	References
Protein stability	RORαK38me1	EZH2	Breast cancer	Inhibition	^60^
	RelAK314me1 K315me1	SET7	MEFs (mouse cardio myocytes), U2OS(osteosarcoma cell), A549(NSCLC cell)	Inhibition	^62^
Nuclear localization	P53K372me1	SET7	293F, U2OS(osteosarcoma cell), H1299(NSCLC cell)	Activation	^64^
	ERαK302me1	SET7	Breast cancer	Activation	^65^
DNA-binding affinity	P53K370me1	SMYD2	H1299(NSCLC cell), U2OS(osteosarcoma cell), BJ-DNp53(fibroblast cell)	Inhibition	^66^
	HIF1αK32me1	SET7	RCC4(renal carcinoma cell)	Inhibition	^70^
	HIF2αK29me1	SET7	RCC4(renal carcinoma cell)	Inhibition	^70^
	P53K382me1	SET8	U2OS(osteosarcoma cell), H1299(NSCLC cell)	Inhibition	^67^
	STAT3 K140me2	SET7	DLD1(colon cancer cell)	Inhibition	^57^
	RelAK37me1	SET7	293T	Activation	^73^
	RelAK218me1, K221me2	NSD1	293C6, HT29(colon cancer cell)	Activation	^74^
	ARK632me*	SET7	Prostate cancer cell	Activation	^72^
	YY1K173me1, K411me1	SET7	HeLa(cervical carcinoma)	Activation	^95^
	YY2 K247me1	SET7	HeLa(cervical carcinoma)	Activation	^96^
Protein–protein binding	RelAK310me1	SETD6	293T, U2OS(osteosarcoma cell), THP1(mononuclear macrophage)	Inhibition	^82^
	RBK860me1	SMYD2	293T, U2OS(osteosarcoma cell), NIH3T3(mouse embryonal fibroblast cell)	Activation	^76^
	RBK873me1	SET7	U2OS(osteosarcoma cell), SAOS2(osteosarcoma cell), C2C12(myoblast), CC42 (fibroblast cell)	Activation	^77^
	P53K382me2	Unknown	U2OS(osteosarcoma cell)	Activation	^75^
Crosstalk with other PTMs	GATA4 K299me1	EZH2	HL1(mouse cardio myocytes)	Inhibition	^97^
	RBK810me1	SET7	U2OS(Osteosarcoma cell)	Inhibition	^81^
	ERαK266me1	SMYD2	Breast cancer	Inhibition	^79^
	STAT3K180me*	EZH2	Glioblastoma	Activation	^80^

Asterisks indicate that the methylation status is unknown

^a^Methylation substrate, lysine site, and methylation degree

^b^Lysine methyl transferases and synonyms: SET7 (KMT7, SET7/9, SET9, SETD7); EZH2 (KMT6A/KMT6); SET8 (PR-Set7, KMT5A, SETD8); NSD1 (KMT3B); SMYD2 (KMT3C)

## Protein stability

Similar to phosphorylation-dependent ubiquitination^58,59^, one study demonstrated that orphan nuclear receptor (RORα) protein stability can be dynamically regulated with methylation-dependent ubiquitination, which is carried out by damage-specific DNA-binding protein 1 (DDB1)/cullin4 (CUL4) E3 ubiquitin ligase complex and a DDB1-CUL4-associated factor 1 (DCAF1) adapter^60^. Methyltransferase EZH2 has been found to methylate RORα at K38. Therefore, monomethylated RORα can be specifically recognized by DCAF1, comprising the putative chromo domain, inducing ubiquitination-dependent degradation through the DCAF1/DDB1/CUL4 axis. Of note, RORα has been proven to be a cancer suppressor^61^. Research has demonstrated an oncogenic role of EZH2 through the facilitation of RORα methylation-dependent degradation, resulting in tumor development and progression^60^.

In addition, previous studies have found that methyltransferase SET7 can methylate DNA-bound RelA (subunit of NF-κB) at lysine residues 314 and 315 in vivo in response to tumor necrosis factor-α (TNFα) stimulation^62^, and the methylation is critical for the degradation of DNA-bound NF-kB and repress NF-kB target genes transcriptional activity.

## Subcellular localization

Nucleocytoplasmic transport is a necessary step for transcription factor activity. Transcription factors modified by phosphorylation can acquire the ability to enter the nucleus^63^. Similarly, lysine methylation can also change nuclear localization and regulate transcriptional activity.

For example, previous research has demonstrated that SET7 specifically methylated p53 at lysine 372, and methylated p53-K372 localized to the nucleus^64^. On the other hand, p53 was shown to be equally distributed between the nuclear and cytosolic fractions. Notably, Chuikov and colleagues showed that p53 stabilization was apparent only in the fraction with chromatin-associated nuclear p53. Given that overexpression of wild-type SET7 resulted in hyper-stabilization and activation of nuclear p53; it could be expected that cell-cycle arrest and apoptosis would result^64^.

Another research study found that the estrogen receptor (ER) could be directly methylated at lysine 302 by SET7. Remarkably, it was found that SET7-mediated methylation enhanced estradiol-induced nuclear accumulation and stability of ER, both of which were necessary for the efficient recruitment of ER to target genes and for subsequent transactivation in breast cancer cells^65^.

## DNA-binding affinity

Lysine methylation changes the binding ability of transcription factors to DNA and regulates their transcriptional activities. The regulatory outcome is related to protein substrate, modification site, and cell context.

### Inhibition of DNA binding

Dimethylation at K140 of signal transducer and activator of transcription 3 (STAT3) by SET7 has been demonstrated to be a negative regulatory event because blockade of this K140 dimethylation greatly increases activated steady-state STAT3 levels and subsequent binding to the promoter of STAT3 target genes^57^.

It has been reported that the methyltransferase SMYD2 could methylate p53 at K370 in cancer cells^66^. The published study by Huang et al. suggests that K370-methylation of p53 reduces DNA-binding efficiency, and SMYD2-mediated methylation at K370 shifts the equilibrium towards dissociation of p53 from DNA^66^. On the other hand, SET7-mediated methylation of p53 at K372 enhances the association of p53 with promoters by blocking SMYD2-mediated methylation of K370, which promotes activation of the target genes^66^. Additionally, another study found that p53 K382me1(lysine 382 monomethylation) generation by the methyltransferase SET8 negatively correlates with DNA damage, and SET8 co-expression reduces the occupancy of p53 at the promoters of the target genes *p21* and *PUMA*^67^.

Hypoxia-inducible factor (HIF)-1 and HIF-2 are the main regulators of cellular responses to hypoxia^68,69^. It has been demonstrated that SET7 methylation of HIF-1 at lysine 32 and HIF-2 at lysine 29 inhibits HIF-1/2 target gene expression by diminishing the occupancy of HIF-1/2 on hypoxia response elements of HIF target gene promoters^70^. These data suggest that SET7-mediated lysine methylation negatively regulates HIF-1/2 transcriptional activity^70^.

### Promotion of DNA binding

Lysine methylation of transcription factors can also enhance DNA-binding affinity. For example, the androgen receptor is a member of the nuclear hormone receptor family of transcription factors that plays a critical role in regulating expression of genes involved in prostate cancer^71^. Methylation of the androgen receptor at lysine 632 by SET7 is necessary for enhancing its transcriptional activity by recruitment to androgen receptor target genes and facilitating inter-domain communication between the N- and C-termini^72^.

NF-κB is a key activator of inflammatory and immune responses with important pathological roles in cancer. SET7 has been found to specifically methylate RelA at lysine 37 with both TNFα and interleukin-1β (IL-1β) treatment^73^. Methylated RelA is restricted to the nucleus and this modification increases its promoter binding affinity. These data suggest that methylation by SET7 enhances the affinity of RelA for DNA, which is a critical event for induction of NF-κB-dependent genes in response to TNFα stimulation. Methylation of K218 and K221 of RelA by the methyltransferase NSD1 plays a positive role in cell proliferation, colony formation, and gene expression in human cancer cells^74^. However, interfering with the expression of NSD1 decreases both NF-κB activity and its ability to bind to DNA in the context of IL-1β treatment.

## Protein–protein interactions

Methylated lysine can be read by specific proteins and linked to specific biological effects on transcriptional activity. For example, dimethylated p53 at lysine 382 is recognized by p53-binding protein 1 (53BP1), which acts as an effector protein^75^. This methylation event can promote the function of p53 in the context of DNA damage.

It has been demonstrated that RB can be methylated at lysine 860 by SMYD2^76^. Furthermore, methylation of RB at K860 provides a direct binding site for the methyl-binding domain of the transcriptional repressor L3MBTL1, which helps to activate the RB function in cancer cells^27^.

In addition, Munro et al. demonstrated that SET7 can methylate lysine 873 of RB both in vitro and in vivo, and methylated RB interacts with heterochromatin protein 1 (HP1)^77^. Furthermore, increases in the levels of bound RB and HP1 on E2F target genes, as measured by chromatin immunoprecipitation, have been observed in conditions of growth arrest. Together, these results reveal that RB and HP1 interact in a SET7-dependent manner, and HP1 contributes to the transcriptional activity of RB^77^.

## Crosstalk with other post-translational modifications

Like ubiquitin and phosphorylation^78^, transcription factor lysine methylation is not limited to a single event. Many studies have found that lysine methylation can achieve distinct biological outcomes indirectly by acting in combination with other types of PMTs that occur at near or distant site^49^.

### Methylation–acetylation crosstalk

It is known that under estrogen-depleted conditions, SMYD2 attenuates chromatin recruitment of ERα to prevent ERα target gene transcriptional activation. Zhang et al. have shown that upon estrogen stimulation, K266 methylation of ERα is diminished^79^. This allows acetyltransferase p300 response element-binding protein to acetylate ERα at K266, thereby promoting ERα transactivation activity. Furthermore, the knockdown of the demethylase LSD1 leads to increased methylation of ERα at K266 and decreased K266/268 acetylation, suggesting that ERα methylation at K266 is dynamically regulated by SMYD2 and LSD1. Taken together, these findings point to a model in which SMYD2 represses ERα target gene expression partly through the inhibition of ERα acetylation at K266/268^79^.

### Methylation–phosphorylation crosstalk

It has been illustrated that EZH2 methylates STAT3 at lysine 180, leading to enhanced STAT3 activity by increasing tyrosine phosphorylation of STAT3^80^. This EZH2–STAT3 interaction preferentially occurs in glioblastoma stem-like cells (GSCs) relative to non-stem tumor cells, and it requires a specific phosphorylation of EZH2^80^.

A study by Carr et al. showed that methylation of RB at K810 by SET7 impedes binding of cyclin-dependent kinases, preventing subsequent phosphorylation of the associated serine residue^81^. This results in retention of RB in the hyperphosphorylated growth suppressing state. In the context of SET7 depletion, RB phosphorylation was not apparent and a reduced expression of E2F target genes, including *DHFR*, *Cdc2,* and *Cdc6*, was seen. Together, the study confirms that SET7 antagonizes cyclin-dependent kinase-dependent cell-cycle progression^81^.

### Transcription factor and histone methylation modification crosstalk

Nuclear RelA monomethylation at K310 by the methyltransferase SETD6 attenuates NF-κB signaling by docking methyltransferase GLP (via its ankyrin repeats) to target genes^82^. This generates a silent chromatin state (H3K9me3), effectively rendering chromatin-bound RelA inert. Therefore, methylation mediated by SETD6 can inhibit RelA target gene expression in an indirect way.

## Biological effects of transcription factor lysine methylation in cancer

**Fig. 3:**
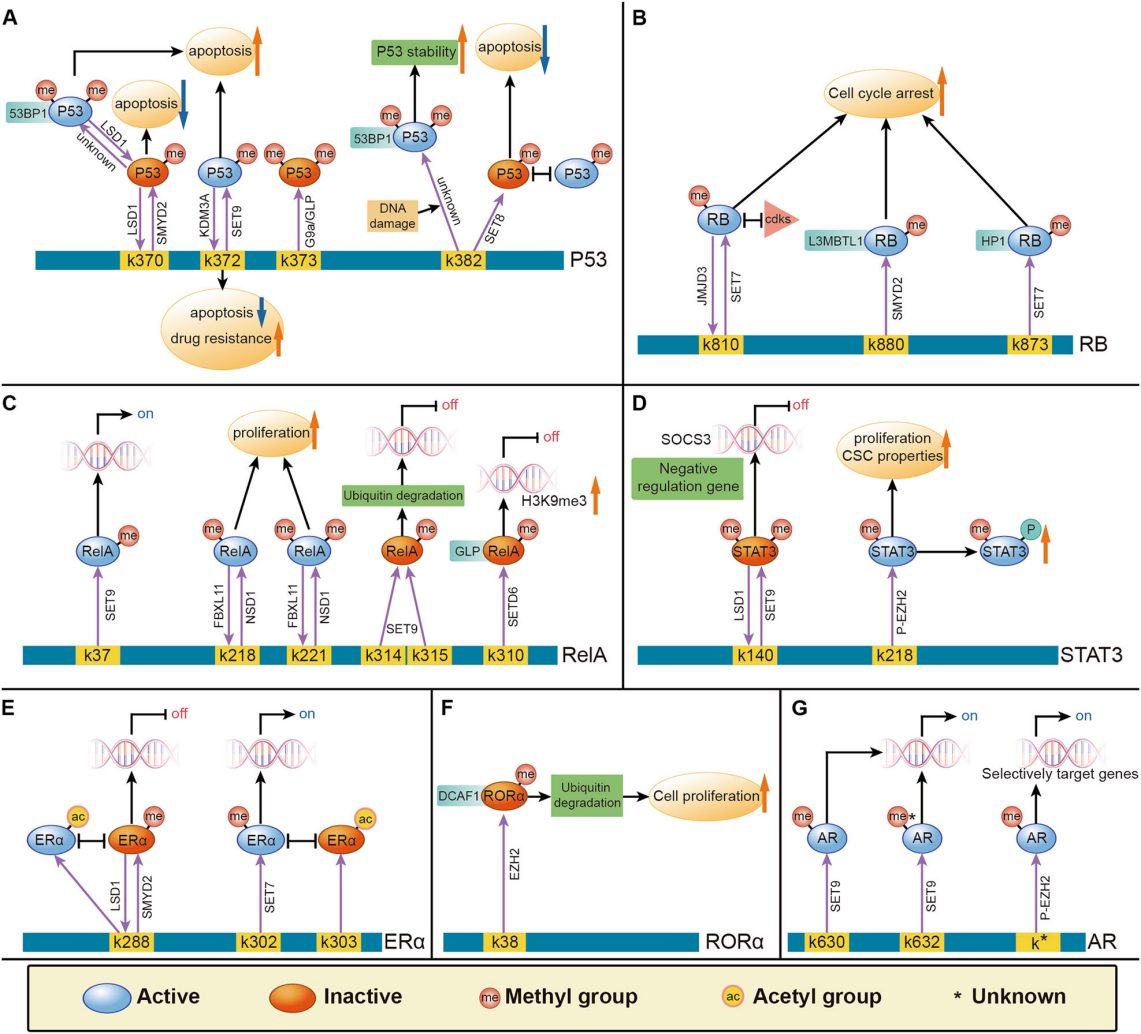
The model of transcription factors in the context of lysine methylation in tumorigenesis. A summary from the current literature of the comprehensive and dynamic methylation processes of transcription factors is depicted in the following models, including of p53 **a**, RB **b**, RelA **c**, STAT3 **d**, ERα **e**, RORα **f**, and AR **g**. Post-translational modifications at specific sites of activate (in blue) and inactivate (in red) transcription factors, thereby affecting regulation of target gene expression specific for cellular biological processes associated with cancer

Lysine methylation is a dynamic process, a small number of transcription factors have been proven to be demethylated by specific KDMs^83^ (Table 2). Herein, we elucidate the comprehensive and dynamic transcription factor methylation processes from the literature and illustrate this summary in models depicted in Fig. 3. Methylation modification at specific sites of transcription factors and the effects on target gene expression and cell biology are shown.

**Table 2:** The known demethylation processes of transcription factors

	Substrate	Enzyme	Tumor type	Transcription activity	References
Demethylation	P53K370me2	LSD1	293T, MCF7 (breast cancer cell), U2OS (Osteosarcoma cell)	Inhibition	^98^
	P53K372me1	KDM3A	Breast cancer	Inhibition	^84^
	RelAK218me1, K221me2	FBXL11	293C6, HT29 (colon cancer cell)	Inhibition	^74^
	ERαK266me1	LSD1	Breast cancer	Activation	^79^
	YY2K247me1	LSD1	HeLa (cervical carcinoma)	Inhibition	^96^

It is noteworthy that several transcription factors that control proliferation, apoptosis, stem cell properties, or drug resistance can be catalyzed by KMTs and KDMs. Unbalanced regulation of these transcription factors plays an important role in the tumor microenvironment, subsequently resulting in cancer initiation and development^36^.

## Proliferation

Recent research has revealed an NF-κB regulatory pathway that is driven by reversible methylation at K218 and K221 of the RelA subunit, carried out by the lysine methyltransferase NSD1 and the lysine demethylase FBXL11^74^. Overexpression of FBXL11 inhibits NF-κB activity, but elevated NSD1 levels can activate NF-κB and reverse the inhibitory effect of FBXL11. The authors also showed that overexpression of FBXL11 slowed the growth of HT29 cancer cells, whereas shRNA-mediated knockdown of FBXL11 had the opposite effect, both of these phenotypes were K218/K221 methylation dependent^74^.

## Apoptosis

Chuikov et al. showed that SET9 can specifically methylate p53 at K372^64^. Methylation of p53 restricts it to the nucleus and increases its stability. Overexpression of the catalytically inactive SET9 was shown to abrogate DNA damage-induced apoptosis, suggesting that the methyltransferase activity of SET9 is critical for induction of p53-dependent apoptosis. This research highlights another possible mechanism for p53 inactivation in human cancers^64^.

On the other hand, SMYD2-mediated methylation of p53 at K370 shifts the equilibrium towards dissociation of p53 from DNA and downregulates expression of *p21* and *MDM2*, thereby inhibiting cell apoptosis^66^.

## Chemotherapy sensitivity

A study by Ramadoss et al. demonstrated that KDM3A suppresses the proapoptotic functions of p53 by removing p53-K372me1^84^. This specific methylation is crucial for the stability of chromatin-bound p53. Unexpectedly, the authors found that inhibition of KDM3A reactivated mutant p53 and induced the expression of proapoptotic genes, thereby restoring apoptotic sensitivity to chemotherapeutic drugs. Taken together, these data suggest that KDM3A might be a potential therapeutic target for human breast cancer treatment and prevention^84^.

## Stem cell properties

Research has shown that EZH2 can methylate STAT3 at K180 in GSCs, which leads to enhanced STAT3 activity by subsequent increases in tyrosine phosphorylation of STAT3^80^. This increased STAT3 activity can contribute to GSC self-renewal and glioblastoma multiforme malignancy.

## Discussion

Lysine methylation of transcription factors is emerging as an important and dynamic PTM to activate or repress target gene expression in response to extracellular signals. Like phosphorylation and acetylation, lysine methylation can directly alter distinct aspects of transcription factor function, including protein stability, cellular localization, DNA-binding affinity, protein–protein interactions, and crosstalk with other PTMs. The biomedical significance of lysine methylation of transcription factors in several human diseases has been explored in recent years. In this review, we summarize the current literature of transcription factor lysine methylation and its role in cancer. We outline the biological significance of this PTM, including effects on proliferation, apoptosis, stem cell properties, and drug resistance in cells, highlighting the importance of transcription factor lysine methylation in carcinogenesis.

Epigenetics provides promising new targets for anticancer therapy^85^. DNA methylation and histone acetylation have been pharmacologically targeted, and several DNA methyltransferase and histone deacetylase inhibitors are FDA-approved for cancer treatment^86,87^. Since methylation is involved in such fundamental cellular functions and is dysregulated in diseases^88^, the investigation of its role in cancer has led to the identification of KMTs and KDMs as promising novel targets for cancer therapy^89–91^. Lysine methylation of transcription factors plays a prominent role in cancer, providing rationale for the development of KMTs and KDMs inhibitors.

Although additional research is required to further understand protein lysine methylation, investigation into inhibitors of methylation regulatory proteins as anticancer drugs is underway and has made considerable progress in recent years^92–94^. Encouragingly, experiments have demonstrated that targeting transcription factor methylation can provide novel therapeutic strategies to target gene mutations and drug resistance in cancer therapy^84^. For example, the lysine-specific demethylase KDM3A has dual carcinogenic effects in breast cancer^84^. By erasing methylation at lysine 9 of histone H3, KDM3A induces preinvasive gene expression. KDM3A can also promote chemotherapy resistance by erasing p53-K372me1. Significantly, depletion of KDM3A is capable of reactivating mutant p53 to induce proapoptotic gene expression. In conclusion, targeting transcription factor methylation can provide new treatment opportunities for overcoming gene mutation and chemotherapeutic resistance in tumors. With the further study of transcription factor lysine methylation, we believe greater clinical therapeutic potential will be explored in the future.

## References

[1] Beato M, Eisfeld K (1997). Transcription factor access to chromatin. Nucleic Acids Res..

[2] Sikder D, Kodadek T (2005). Genomic studies of transcription factor-DNA interactions. Curr. Opin. Chem. Biol..

[3] Eckersley-Maslin MA, Alda-Catalinas C, Reik W (2018). Dynamics of the epigenetic landscape during the maternal-to-zygotic transition. Nat. Rev. Mol. Cell Biol..

[4] Dias S (2017). Effector regulatory T cell differentiation and immune homeostasis depend on the transcription factor Myb. Immunity.

[5] Kang J, Malhotra N (2015). Transcription factor networks directing the development, function, and evolution of innate lymphoid effectors. Annu. Rev. Immunol..

[6] Brown MS, Goldstein JL (1997). The SREBP pathway: regulation of cholesterol metabolism by proteolysis of a membrane-bound transcription factor. Cell.

[7] Vizcaino C, Mansilla S, Portugal J (2015). Sp1 transcription factor: a long-standing target in cancer chemotherapy. Pharmacol. Ther..

[8] Cao J (2018). Twist promotes tumor metastasis in basal-like breast cancer by transcriptionally upregulating ROR1. Theranostics.

[9] Kaypee S (2016). Aberrant lysine acetylation in tumorigenesis: implications in the development of therapeutics. Pharmacol. Ther..

[10] Filtz TM, Vogel WK, Leid M (2014). Regulation of transcription factor activity by interconnected post-translational modifications. Trends Pharmacol. Sci..

[11] Wan J (2016). PCAF-mediated acetylation of transcriptional factor HOXB9 suppresses lung adenocarcinoma progression by targeting oncogenic protein JMJD6. Nucleic Acids Res..

[12] Anders L (2011). A systematic screen for CDK4/6 substrates links FOXM1 phosphorylation to senescence suppression in cancer cells. Cancer Cell.

[13] Carr SM, Poppy Roworth A, Chan C, La Thangue NB (2015). Post-translational control of transcription factors: methylation ranks highly. FEBS J..

[14] Bannister AJ, Miska EA (2000). Regulation of gene expression by transcription factor acetylation. Cell. Mol. Life Sci..

[15] Whitmarsh AJ, Davis RJ (2000). Regulation of transcription factor function by phosphorylation. Cell. Mol. Life Sci..

[16] Wang Q, Wang K, Ye M (2017). Strategies for large-scale analysis of non-histone protein methylation by LC-MS/MS. Analyst.

[17] Biggar KK, Li SS (2015). Non-histone protein methylation as a regulator of cellular signalling and function. Nat. Rev. Mol. Cell Biol..

[18] Hamamoto R, Saloura V, Nakamura Y (2015). Critical roles of non-histone protein lysine methylation in human tumorigenesis. Nat. Rev. Cancer.

[19] Binda O (2013). On your histone mark, SET, methylate!. Epigenetics.

[20] Biggar KK, Wang Z, Li SS (2017). SnapShot: lysine methylation beyond histones. Mol. Cell.

[21] Wang ZA, Liu WR (2017). Proteins with site-specific lysine methylation. Chemistry.

[22] Lanouette S, Mongeon V, Figeys D, Couture JF (2014). The functional diversity of protein lysine methylation. Mol. Syst. Biol..

[23] Mozzetta C, Boyarchuk E, Pontis J, Ait-Si-Ali S (2015). Sound of silence: the properties and functions of repressive Lys methyltransferases. Nat. Rev. Mol. Cell Biol..

[24] Dillon SC, Zhang X, Trievel RC, Cheng X (2005). The SET-domain protein superfamily: protein lysine methyltransferases. Genome Biol..

[25] Petrossian TC, Clarke SG (2011). Uncovering the human methyltransferasome. Mol. Cell. Proteom..

[26] Cho HS (2012). Enhanced HSP70 lysine methylation promotes proliferation of cancer cells through activation of Aurora kinase B. Nat. Commun..

[27] Stark GR, Wang Y, Lu T (2011). Lysine methylation of promoter-bound transcription factors and relevance to cancer. Cell Res..

[28] Suzuki T, Terashima M, Tange S, Ishimura A (2013). Roles of histone methyl-modifying enzymes in development and progression of cancer. Cancer Sci..

[29] Selvi B, Mohankrishna D, Ostwal Y, Kundu T (2010). Small molecule modulators of histone acetylation and methylation: a disease perspective. Biochim. Biophys. Acta.

[30] Kaniskan HU, Jin J (2017). Recent progress in developing selective inhibitors of protein methyltransferases. Curr. Opin. Chem. Biol..

[31] Alam H, Gu B, Lee MG (2015). Histone methylation modifiers in cellular signaling pathways. Cell. Mol. Life Sci..

[32] Lu T (2013). Role of lysine methylation of NF-kappaB in differential gene regulation. Proc. Natl Acad. Sci. USA.

[33] Black JC, Van Rechem C, Whetstine JR (2012). Histone lysine methylation dynamics: establishment, regulation, and biological impact. Mol. Cell.

[34] Colon-Bolea P, Crespo P (2014). Lysine methylation in cancer: SMYD3-MAP3K2 teaches us new lessons in the Ras-ERK pathway. Bioessays.

[35] Greer EL, Shi Y (2012). Histone methylation: a dynamic mark in health, disease and inheritance. Nat. Rev. Genet..

[36] Morera L, Lubbert M, Jung M (2016). Targeting histone methyltransferases and demethylases in clinical trials for cancer therapy. Clin. Epigenetics.

[37] Falnes PO, Jakobsson ME, Davydova E, Ho A, Malecki J (2016). Protein lysine methylation by seven-beta-strand methyltransferases. Biochem. J..

[38] Shi Y (2004). Histone demethylation mediated by the nuclear amine oxidase homolog LSD1. Cell.

[39] Lei ZJ (2015). Lysine-specific demethylase 1 promotes the stemness and chemoresistance of Lgr5(+) liver cancer initiating cells by suppressing negative regulators of beta-catenin signaling. Oncogene.

[40] Karytinos A (2009). A novel mammalian flavin-dependent histone demethylase. J. Biol. Chem..

[41] Klose RJ, Kallin EM, Zhang Y (2006). JmjC-domain-containing proteins and histone demethylation. Nat. Rev. Genet..

[42] Ambler RP, Rees MW (1959). Epsilon-N-methyl-lysine in bacterial flagellar protein. Nature.

[43] Murray K (1964). The occurrence of epsilon-N-methyl lysine in histones. Biochemistry.

[44] Moore KE (2013). A general molecular affinity strategy for global detection and proteomic analysis of lysine methylation. Mol. Cell.

[45] Theillet FX (2012). Site-specific mapping and time-resolved monitoring of lysine methylation by high-resolution NMR spectroscopy. J. Am. Chem. Soc..

[46] van Loosdregt J, Coffer PJ (2014). Post-translational modification networks regulating FOXP3 function. Trends Immunol..

[47] Everett LJ, Jensen ST, Hannenhalli S (2011). Transcriptional regulation via TF-modifying enzymes: an integrative model-based analysis. Nucleic Acids Res..

[48] Zhang X, Huang Y, Shi X (2015). Emerging roles of lysine methylation on non-histone proteins. Cell. Mol. life Sci..

[49] Wu Z, Connolly J, Biggar KK (2017). Beyond histones: the expanding roles of protein lysine methylation. FEBS J..

[50] Holmberg CI, Tran SE, Eriksson JE, Sistonen L (2002). Multisite phosphorylation provides sophisticated regulation of transcription factors. Trends Biochem. Sci..

[51] Geiss-Friedlander R, Melchior F (2007). Concepts in sumoylation: a decade on. Nat. Rev. Mol. Cell Biol..

[52] Conaway RC, Brower CS, Conaway JW (2002). Emerging roles of ubiquitin in transcription regulation. Science.

[53] Huang J (2010). G9a and Glp methylate lysine 373 in the tumor suppressor p53. J. Biol. Chem..

[54] Ko S (2011). Lysine methylation and functional modulation of androgen receptor by Set9 methyltransferase. Mol. Endocrinol..

[55] Xu K (2012). EZH2 oncogenic activity in castration-resistant prostate cancer cells is polycomb-independent. Science.

[56] Pless O (2008). G9a-mediated lysine methylation alters the function of CCAAT/enhancer-binding protein-beta. J. Biol. Chem..

[57] Yang J (2010). Reversible methylation of promoter-bound STAT3 by histone-modifying enzymes. Proc. Natl Acad. Sci. USA.

[58] Sun SC (2012). The noncanonical NF-kappaB pathway. Immunol. Rev..

[59] Wu RC, Feng Q, Lonard DM, O’Malley BW (2007). SRC-3 coactivator functional lifetime is regulated by a phospho-dependent ubiquitin time clock. Cell.

[60] Lee JM (2012). EZH2 generates a methyl degron that is recognized by the DCAF1/DDB1/CUL4 E3 ubiquitin ligase complex. Mol. Cell.

[61] Lee JM (2010). RORalpha attenuates Wnt/beta-catenin signaling by PKCalpha-dependent phosphorylation in colon cancer. Mol. Cell.

[62] Yang XD (2009). Negative regulation of NF-kappaB action by Set9-mediated lysine methylation of the RelA subunit. EMBO J..

[63] Wen Z, Zhong Z, Darnell JE (1995). Maximal activation of transcription by Stat1 and Stat3 requires both tyrosine and serine phosphorylation. Cell.

[64] Chuikov S (2004). Regulation of p53 activity through lysine methylation. Nature.

[65] Subramanian K (2008). Regulation of estrogen receptor alpha by the SET7 lysine methyltransferase. Mol. Cell.

[66] Huang J (2006). Repression of p53 activity by Smyd2-mediated methylation. Nature.

[67] Shi X (2007). Modulation of p53 function by SET8-mediated methylation at lysine 382. Mol. Cell.

[68] Semenza GL (2013). HIF-1 mediates metabolic responses to intratumoral hypoxia and oncogenic mutations. J. Clin. Investig..

[69] Pugh CW, Ratcliffe PJ (2003). Regulation of angiogenesis by hypoxia: role of the HIF system. Nat. Med..

[70] Liu X (2015). Repression of hypoxia-inducible factor α signaling by Set7-mediated methylation. Nucleic Acids Res..

[71] Han Y (2017). Triptolide inhibits the AR signaling pathway to suppress the proliferation of enzalutamide resistant prostate cancer cells. Theranostics.

[72] Gaughan L (2011). Regulation of the androgen receptor by SET9-mediated methylation. Nucleic Acids Res..

[73] Ea CK, Baltimore D (2009). Regulation of NF-kappaB activity through lysine monomethylation of p65. Proc. Natl Acad. Sci. USA.

[74] Lu T (2010). Regulation of NF-kappaB by NSD1/FBXL11-dependent reversible lysine methylation of p65. Proc. Natl Acad. Sci. USA.

[75] Kachirskaia I (2008). Role for 53BP1 Tudor domain recognition of p53 dimethylated at lysine 382 in DNA damage signaling. J. Biol. Chem..

[76] Saddic LA (2010). Methylation of the retinoblastoma tumor suppressor by SMYD2. J. Biol. Chem..

[77] Munro S, Khaire N, Inche A, Carr S, La Thangue NB (2010). Lysine methylation regulates the pRb tumour suppressor protein. Oncogene.

[78] Zhao Y, Brickner JR, Majid MC, Mosammaparast N (2014). Crosstalk between ubiquitin and other post-translational modifications on chromatin during double-strand break repair. Trends Cell Biol..

[79] Zhang X (2013). Regulation of estrogen receptor alpha by histone methyltransferase SMYD2-mediated protein methylation. Proc. Natl Acad. Sci. USA.

[80] Kim E (2013). Phosphorylation of EZH2 activates STAT3 signaling via STAT3 methylation and promotes tumorigenicity of glioblastoma stem-like cells. Cancer Cell.

[81] Carr SM, Munro S, Kessler B, Oppermann U, La Thangue NB (2011). Interplay between lysine methylation and Cdk phosphorylation in growth control by the retinoblastoma protein. EMBO J..

[82] Levy D (2011). Lysine methylation of the NF-kappaB subunit RelA by SETD6 couples activity of the histone methyltransferase GLP at chromatin to tonic repression of NF-kappaB signaling. Nat. Immunol..

[83] Kooistra S, Helin K (2012). Molecular mechanisms and potential functions of histone demethylases. Nat. Rev. Mol. Cell Biol..

[84] Ramadoss S, Guo G, Wang C (2017). Lysine demethylase KDM3A regulates breast cancer cell invasion and apoptosis by targeting histone and the non-histone protein p53. Oncogene.

[85] Zheng Y, Wu J, Chen Z, Goodman M (2008). Chemical regulation of epigenetic modifications: opportunities for new cancer therapy. Med. Res. Rev..

[86] Pfister SX, Ashworth A (2017). Marked for death: targeting epigenetic changes in cancer. Nat. Rev. Drug Discov..

[87] Schapira M, Arrowsmith CH (2016). Methyltransferase inhibitors for modulation of the epigenome and beyond. Curr. Opin. Chem. Biol..

[88] Yu Y (2013). High expression of lysine-specific demethylase 1 correlates with poor prognosis of patients with esophageal squamous cell carcinoma. Biochem. Biophys. Res. Commun..

[89] Tian X (2013). Histone lysine-specific methyltransferases and demethylases in carcinogenesis: new targets for cancer therapy and prevention. Curr. Cancer Drug Targets.

[90] Kaniskan HU, Martini ML, Jin J (2018). Inhibitors of protein methyltransferases and demethylases. Chem. Rev..

[91] Kondengaden S (2016). Discovery of novel small molecule inhibitors of lysine methyltransferase G9a and their mechanism in leukemia cell lines. Eur. J. Med. Chem..

[92] Knutson SK (2014). Selective inhibition of EZH2 by EPZ-6438 leads to potent antitumor activity in EZH2-mutant non-Hodgkin lymphoma. Mol. Cancer Ther..

[93] Barsyte-Lovejoy D (2014). (R)-PFI-2 is a potent and selective inhibitor of SETD7 methyltransferase activity in cells. Proc. Natl Acad. Sci. USA.

[94] Greiner D, Bonaldi T, Eskeland R, Roemer E, Imhof A (2005). Identification of a specific inhibitor of the histone methyltransferase SU(VAR)3-9. Nat. Chem. Biol..

[95] Zhang WJ (2016). Regulation of transcription factor Yin Yang 1 by SET7/9-mediated lysine methylation. Sci. Rep..

[96] Wu XN (2017). Methylation of transcription factor YY2 regulates its transcriptional activity and cell proliferation. Cell Discov..

[97] He A (2012). PRC2 directly methylates GATA4 and represses its transcriptional activity. Genes Dev..

[98] Huang J (2007). p53 is regulated by the lysine demethylase LSD1. Nature.

